# Obituary

**Published:** 1912-03

**Authors:** 


BUFFALO has lost during the last month, two distinguished laymen whose work and influence have borne directly on our profession. Hon. J. N. Adam, has left, among other gifts to his townsmen, the Tuberculosis Hospital at Perrysburg. T. Guilford Smith, LL.D., beside participating in the direction of several medical institutions, was for several years on the State Board of Regents and in this capacity, had an active part in elevating the standards of medical education.

